# Acetaminophen Interactions with Phospholipid Vesicles Induced Changes in Morphology and Lipid Dynamics

**DOI:** 10.1021/acs.langmuir.1c01458

**Published:** 2021-07-30

**Authors:** Judith U. De Mel, Sudipta Gupta, Sydney Harmon, Laura Stingaciu, Eric W. Roth, Miriam Siebenbuerger, Markus Bleuel, Gerald J. Schneider

**Affiliations:** † Department of Chemistry, Louisiana State University, Baton Rouge, Louisiana 70803, United States; ‡ Department of Chemistry, Colorado School of Mines, Golden, Colorado 80401, United States; § Neutron Sciences Directorate, Oak Ridge National Laboratory (ORNL), P.O.B 2008, 1 Bethel Valley Road, Oak Ridge, Tennessee 37831, United States; ∥ Department of Materials Science and Engineering and NUANCE Center, Northwestern University, 2220 Campus Drive, Evanston, Illinois 60208, United States; ⊥ Center of Advanced Microstructures and Devices, Louisiana State University, 6980 Jefferson Highway, Baton Rouge, Louisiana 70806, United States; # NIST Center for Neutron Research, National Institute of Standards and Technology, Gaithersburg, Maryland 20899-8562, United States; ¶ Department of Physics & Astronomy, Louisiana State University, Baton Rouge, Louisiana 70803, United States

## Abstract

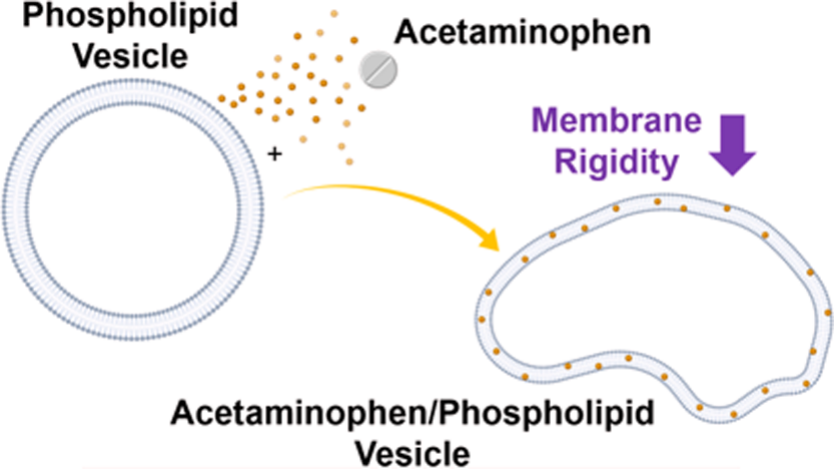

Acetaminophen (APAP) or paracetamol, despite its wide and common use for pain and fever symptoms, shows a variety of side effects, toxic effects, and overdose effects. The most common form of toxic effects of APAP is in the liver where phosphatidylcholine is the major component of the cell membrane with additional associated functionalities. Although this is the case, the effects of APAP on pure phospholipid membranes have been largely ignored. Here, we used 1,2-di-(octadecenoyl)-*sn*-glycero-3-phosphocholine (DOPC), a commonly found phospholipid in mammalian cell membranes, to synthesize large unilamellar vesicles to investigate how the incorporation of APAP changes the pure lipid vesicle structure, morphology, and fluidity at different concentrations. We used a combination of dynamic light scattering, small-angle neutron and X-ray scattering (SANS, SAXS), and cryo-TEM for structural characterization, and neutron spin-echo (NSE) spectroscopy to investigate the dynamics. We showed that the incorporation of APAP in the lipid bilayer significantly impacts the spherical phospholipid self-assembly in terms of its morphology and influences the lipid content in the bilayer, causing a decrease in bending rigidity. We observe a decrease in the number of lipids per vesicle by almost 28% (0.06 wt % APAP) and 19% (0.12 wt % APAP) compared to the pure DOPC (0 wt % APAP). Our results showed that the incorporation of APAP reduces the membrane rigidity by almost 50% and changes the spherical unilamellar vesicles into much more irregularly shaped vesicles. Although the bilayer structure did not show much change when observed by SAXS, NSE and cryo-TEM results showed the lipid dynamics change with the addition of APAP in the bilayer, which causes the overall decreased membrane rigidity. A strong effect on the lipid tail motion showed that the space explored by the lipid tails increases by a factor of 1.45 (for 0.06 wt % APAP) and 1.75 (for 0.12 wt % APAP) compared to DOPC without the drug.

## Introduction

Acetaminophen (APAP) and popular NSAIDs (nonsteroidal anti-inflammatory drugs) such as aspirin (ASA), and ibuprofen (IBU) have been used for decreasing inflammation and pain relief for centuries. Commercially available from the 1950s, many over-the-counter anti-inflammatory and pain medications are extensively used worldwide without prescription control. Among these, APAP is the most commonly used, which has a profound presence in antipyretic and analgesic usage that it is almost to a point of overuse.^1^ The danger arises due to the toxic effects and side effects on mammals these drugs can have.^2−6^ It has also been shown that despite the low dosages consumed, the drugs tend to accumulate and concentrate in different tissues where the therapeutic effects, side effects, and toxic effects are detected. Therefore, researchers from various disciplines have attempted to unravel their mechanisms of actions in humans and other animal and tissue models using a variety of approaches.

APAP or paracetamol is considered the first-line choice for pain relief, while drugs such as ASA and IBU are considered anti-inflammatory counterparts.^7^ Despite the differences in application, one thing these drugs have in common is that their main mechanism of action is connected to a membrane-bound protein family called cyclooxygenase (COX), which regulates prostaglandin formation, which then in turn regulates inflammatory responses and pain.^8^ Over the years, the focus of understanding the underlying mechanisms of actions of APAP has been in connection to the COX enzyme centers and other related proteins. Despite the long history of use in modern medicine, mechanisms of action of APAP are complicated and not completely understood.^9−12^ Although this is the case, APAP is currently known as a COX inhibitor by competitive inhibition to the active site, which binds arachidonic acid,^9^ as opposed to the action of other drugs such as IBU, which is related to non-specific inhibition of COX,^13^ or ASA, which has shown activity related to chemopreventive effects and platelet aggregation in addition to being a COX inhibitor by covalently modifying COX active sites.^14^ Therefore, despite the common relationship with the COX enzymes, these drugs have diverse interaction pathways, which calls for an independent investigation of the impacts of the drugs to understand their unique effects. One aspect that requires continuous attention is the drug–lipid membrane interactions. Studies have shown different impacts of small drug molecules on lipid membranes such as induced fusion,^15^ membrane permeability,^16,17^ and changes in membrane rigidity.^18−20^ Particularly, drug-induced membrane rigidity changes are important to explore due to the relationship of membrane rigidity with multiple functions such as the metastatic potential of cancer cells,^21−23^ apoptosis,^24^ erythrocyte morphology,^25^ and so on.

**Figure 1:**
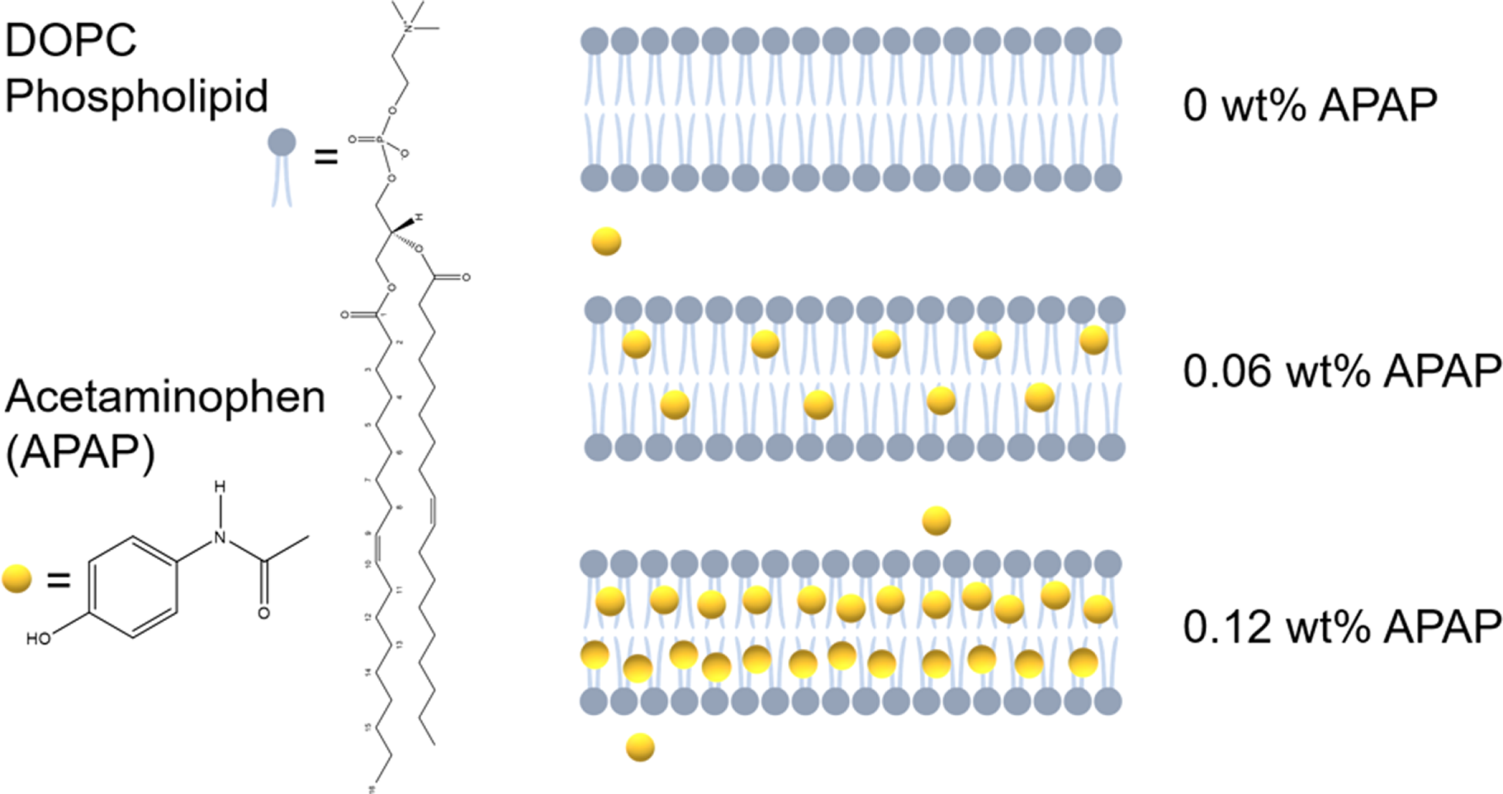
Chemical structures of the DOPC phospholipid and APAP on the left and the schematic representation of the three scenarios of gradual incorporation of APAP at 0, 0.06, and 0.12 wt % explored on the right.

APAP is a small drug molecule (C_8_H_9_NO_2_, 151.1626 g mol^–1^) that consists of an aromatic core with an acetanilide functional group and a hydroxyl functional group in para position to each other. The p*K*_a_ value of 9.38 renders the molecule to be charge-neutral in physiological pH as illustrated in Figure 1.^26^ Molecular dynamics simulations predict that APAP molecules are located close to the carbonyl group region of the phospholipids in an intermediate location between the hydrophobic and hydrophilic parts of the phospholipid.^27^ Understanding the unique effects of APAP on mammalian cells particularly, molecular-level details of the drug’s influences on physicochemical properties of the cell membrane integrity and fluidity are critical for future therapeutic advancements (e.g., decreasing side-effects due to toxicity). APAP overdose effects have also been researched extensively.^28^ The most common form of APAP toxicity occurs in the liver where phosphatidylcholine (PC) is the primary component of the cell membrane in addition to its other functions such as being a precursor of signaling molecules as well as being a key element in lipoprotein and bile.^29^ Many studies have shown a connection between phospholipids with the APAP activity. Bhattacharyya et al. showed the presence of PCs and lysoPCs with very long fatty acids significantly decreased in overdose conditions, indicative of a structure–activity relationship with enzymes responsible for the phospholipid metabolism.^30^ Recently, Yamada et al. showed mechanisms where acute liver failure induced by APAP toxicity is ferroptosis-mediated, which is driven by polyunsaturated fatty acids.^31^ Ming et al. have also reported that the APAP overdose conditions induce dramatic changes to the PC and phosphatidylethanolamine profiles of plasma and liver through possible hepatocyte damage and interferences to the phospholipid metabolism.^29^ Therefore, the importance of investigating APAP effects on pure phospholipid bilayer structures becomes an important research question from a fundamental, material, and chemical standpoint.

Here, we have investigated the structural and dynamic details of drug–lipid vesicle interactions in the hope of broadening the knowledge of biophysical processes that may carry links to many long-standing questions in the realm of the drug’s side-effects and toxicity effects at the molecular basis. We hypothesized that the contribution from the incorporated APAP to the phospholipid bilayer is enough to contribute to changes in physicochemical properties of the bilayer, the morphology of the vesicle self-assembly, and lipid dynamics at nanometer length-scale and nanosecond timescale, which will result in functional changes to the membrane-bound enzymes, and so on, regulating APAP mechanisms of action and its side/overdose effects. To investigate these structural, morphological, and dynamical changes, we used 1,2-di-(octadecenoyl)-*sn*-glycero-3-phosphocholine (DOPC) large unilamellar vesicles with APAP (Figure 1) and conducted dynamic light scattering (DLS), small-angle X-ray and neutron scattering (SAXS and SANS), cryo-transmission electron microscopy (cryo-TEM), and neutron spin-echo (NSE) spectroscopy studies.

## Theoretical Background

### Vesicle Structure

The vesicle form factor is modeled using an extension of the core–shell model used in our previous studies.^32−36^ For unilamellar vesicles, the core–shell model consists of a water core of radius *R*_c_, encapsulated by three shells with (i) a lipid inner-head, (ii) a tail region (hydrocarbon core), and (iii) an outer head. The 1D scattering pattern is given by
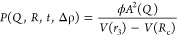
1where ϕ is the lipid volume fraction. The scattering contributions from three different shells to obtain the vesicle form factor, *P*(*Q*,*R*,*t*,Δρ), are described in detail in the Supporting Information. Here, Δρ is the neutron contrast calculated from the neutron scattering length densities of the lipid bilayer of thickness, *t*, and vesicle radius, *R*. Here, The macroscopic scattering cross section is obtained by
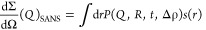
2For the size polydispersity, *s*(*r*), we used a log-normal distribution.^37^

A Gaussian distribution was used to include the polydispersity of the bilayer and the water layer in the case of multilamellar vesicles.

### Bilayer Structure

To get direct access to the macroscopic scattering cross section given by the SAXS scattering intensity from a random lamellar sheet consisting of lipid heads and tails of thicknesses are δ_H_ and δ_T_, respectively, given by
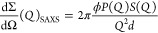
3with particle volume fraction, ϕ, and the lamellar repeat distance, *d*. The details of the bilayer form factor, *P*(*Q*), and the inter-bilayer structure factors are given in the Supporting Information. A Gaussian distribution function includes thickness polydispersity for *d*, δ_H_, and δ_T_.

### Dynamics of a Lipid Bilayer

The overall membrane dynamics can be a result of the superposition of different motions over different lengths and time scales. NSE spectroscopy has been vital to understanding the membrane fluctuations at the length scale of the lipid-bilayer thickness.^33,35,38−41^ The dynamic structure factor or the intermediate scattering function, *S*(*Q*,*t*), from NSE can be modeled assuming statistically independent tail-motion, height–height fluctuations (membrane undulations), and translational diffusion (of the entire vesicle)^38^
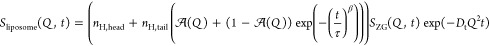
4

Here, the relative fractions of protons in the head are kept fixed to *n*_H,head_ = 0.21 for h-DOPC and *n*_H,tail_ = 1 – *n*_H,head_ = 0.79. The variable  represents the elastic fraction of the lipid tail motion. The exponential term represents the membrane undulation within the Zilman–Granek (ZG) model^42^
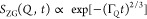
5The *Q*-dependent decay rate, Γ_*Q*_, can be used to determine the intrinsic bending modulus, *κ*_η_, by^43−45^
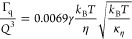
6Here, η is the viscosity, *k*_B_ is the Boltzmann constant, and *T* is the temperature. For lipid bilayers, *γ* = 1.^40−42,44−46^

To obtain model-independent data, we analyze the mean squared displacement (Δ*r*(*t*)^2^, MSD) and the non-Gaussianity parameter, , from the measured dynamic structure factor, *S*(*Q*,*t*), using a cumulant expansion given by^33,38,47,48^
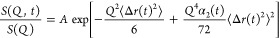
7The non-Gaussianity parameter α_2_ is essentially defined as a function of the fourth ⟨Δ*r*(*t*)^4^⟩ and the second moment squared ⟨Δ*r*(*t*)^2^⟩^2^. Here, we use the space dimension, *d* = 3.^33,48,49^

An alternative representation of eqs 5 and 6 is^38^
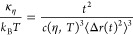
8with . Equation 8 is strictly valid within the ZG approximation ⟨Δ*r*(*t*)^2^⟩ ∝ *t*^2/3^. Any deviation indicates dynamics beyond ZG undulations.

## Experimental Section

### Materials

All chemicals and reagents were used as received. DOPC (MC 8181, Lot No. 1905811L) was purchased from NOF America Corporation (White Plains, NY, USA). APAP powder, USP (Lot No. 2HE0496) was purchased from Spectrum Chemical MFG CORP (New Brunswick, NY, USA). APAP powder was kept in the freezer (−20 °C) in dry-dark conditions until use due to its light sensitivity. All other organic reagents (HPLC grade) and D_2_O (99.8% deuterated) were received from Sigma Aldrich (St. Louis, MO, USA).

### Sample Preparation

DOPC lyophilized powder was dissolved in chloroform (40 mg/mL) and APAP in isopropanol (4.8 mg/mL). Then, DOPC and APAP dissolved in the respective organic solvents were mixed to obtain specific weight ratios: for 1 wt % DOPC 0 wt % APAP 1 mL of DOPC/chloroform was used and for 1 wt % DOPC with 0.06 wt % APAP and 0.12 wt % APAP, 1 mL of DOPC/chloroform solution with 0.5 mL of APAP/isopropanol and 1 mL of APAP/isopropanol were mixed, respectively. A rotary evaporator and vacuum oven (overnight) were used to remove the organic solvent traces. The obtained dry lipid cakes were hydrated with D_2_O (4 mL each). The vesicle suspensions were then subjected to 10 freeze–thaw cycles (−20 °C/50 °C) in 10 min intervals. Finally, the vesicles were extruded using an Avanti mini-extruder with 100 nm polycarbonate membranes passing the vesicle suspension 33 times through the membrane to obtain large unilamellar vesicles of ∼100 nm. The final DOPC concentrations of the samples were 1 wt % with the APAP concentrations being 0 wt % (pure DOPC), 0.06, and 0.12 wt %. Molar ratios of DOPC/APAP for the samples were 1:0, 3:1, and 5:3. The same sample concentration of DOPC was used for all the studies (DLS, SANS, SAXS, cryo-TEM, and NSE). The corresponding sample volumes were 0.5 mL (DLS), 0.45 mL (SANS), 40 μL (SAXS), 4 μL (cryo-TEM), and 3.5 mL (NSE).

All measurements were conducted at a 1 wt % DOPC concentration at ambient temperature of 20–25 °C where the DOPC lipid was in the fluid phase. Results shown in this MS are samples prepared on two different occasions due to the different dates of accessibility to the respective instruments. [Batch 1: DLS, SANS, SAXS (at Stanford Synchrotron) and NSE] [Batch 2: cryo-TEM and SAXS (at LSU-CAMD)]. However, the same procedure was followed. The samples and data were reproducible within experimental accuracy (see the Supporting Information).

### Dynamic Light Scattering

DLS experiments were conducted at the LSU Polymer Analysis Laboratory (LSU PAL) using a Malvern Zetasizer Nano ZS instrument. (Specifications: He–Ne laser wavelength, λ = 633 nm at 30 mW laser power, backscattering set up angle θ = 173°). A 0.5 mL aliquot of each sample was added to a disposable micro-UV cuvette and the respective DLS data were obtained using D_2_O as the solvent at 25 °C. The hydrodynamic radius, *R*_h_, of the liposomes in pure DOPC vesicles and each of the DOPC/APAP vesicles were calculated from the translational diffusion coefficient, *D*_t_, using the Stokes–Einstein equation, *R*_h_ = *k*_B_*T*/(6πη_0_*D*_t_), with the Boltzmann constant, *k*_B_, the temperature, *T*, and the viscosity of the solvent, η_0_. DLS measurements were triplicated for each sample and averaged. The results are listed in Table 1.

**Table 1:** Parameters Found from SANS and DLS in Figure 2^a^

APAP concentration (wt %)	b*R*_SANS_ (nm)	bδ_HH_ (nm)	b*N*_agg_ × 10^4^	bPD_SANS_ %	c*R*_h_ (nm) _DLS_	cPD_DLS_ %
0.00	51.2 ± 0.4	3.9 ± 0.1	9.3 ± 0.2	27	61.6 ± 0.2	37
0.06	43.5 ± 0.6	4.0 ± 0.3	6.7 ± 0.7	27	59.5 ± 0.2	38
0.12	46.4 ± 0.5	3.9 ± 0.2	7.5 ± 0.5	27	60.1 ± 0.3	44

a *R*_SANS_ vesicle radius from SANS, δ_HH_ is the lipid head-to-head distance, *N*_agg_ is the aggregation number depicting the number of lipids per liposome, PD is the polydispersity percentage, and *R*_h_ is the hydrodynamic radius of the vesicles found from the log-normal fitting of the size distribution.

b By SANS core–shell model.

c By log-normal size distribution fit.

### Cryo-Transmission Electron Microscopy

The cryo-TEM data were obtained at the BioCryo facility of the Northwestern University Nuance Center (remote access due to COVID19 restrictions). The samples were applied to 200 mesh Cu grids with a lacey carbon membrane. Before plunge-freezing, grids (EMS Cat# LC200-CU-100) were glow discharged in a Pelco easiGlow glow discharger (Ted Pella Inc., Redding, CA, USA) using an atmosphere plasma generated at 15 mA for 15 s with a pressure of 0.24 mbar. This treatment created a negative charge on the carbon membrane, allowing liquid samples to spread evenly over the grid. A 4 μL volume of each sample was pipetted onto the grid and blotted for 5 s with a blot offset of +0.5 mm, followed by immediate plunging into liquid ethane within an FEI Vitrobot Mark III plunge freezing instrument (Thermo Fisher Scientific, Waltham, MA, USA). Grids were then transferred to liquid nitrogen for storage. The plunge-frozen grids were kept vitreous at −180 °C in a Gatan Cryo Transfer Holder model 626.6 (Gatan Inc., Pleasanton, CA, USA) while viewing using a Hitachi HT7700 W-emission transmission electron microscope at 100 kV. Image data were collected by a Gatan Orius 4k × 2.67k digital camera (Gatan Inc., Pleasanton, CA, USA). Samples required no dilution at 1 wt % concentration for successful visualization. Cryo-TEM images were analyzed using ImageJ software.

### Small-Angle X-ray Scattering

SAXS data were obtained on two occasions. SAXS results displayed in Figure 3 were obtained at the Stanford Synchrotron radiation light source (SSRL), beamline 4-2.^50^ An automated sampler system was used to load 40 μL of aliquots of samples to the capillary cell where the sample was exposed to X-ray radiation 24 times in 1 s exposures as the flow cell gently moves back and forth to minimize any potential radiation damage by continuous spot exposure. The scans were then statistically averaged, and radial averaging over the same data yields the intensity as a function of the momentum transfer. The data were obtained at a beam energy of 11 keV at the detector distance of 1 m to explore a *Q* range of 0.01–1 Å^–1^ using a Pilatus 3 × 1 M detector. Data reduction was done using standard SSRL protocols implemented in the software Blu-Ice.^51^ SAXS data obtained at the Louisiana State University Center for Advanced Microstructures and Devices (CAMD) are listed in the Supporting Information. The CAMD SAXS/WAXS/GISAXS beamline at LSU, Baton Rouge, Louisiana, was manufactured by Saxslab, now Xenocs (France, USA) by including the pre-existing sample chamber of the former SAXS beamline. The experiments were performed in lab mode with a Genix Cu K_α_ lab source (Xenocs). A Pilatus 3R 300K detector was placed outside the evacuated flight tube at a sample to detector distance of 263.40 mm. In order to avoid additional windows in the flight path, the sample chamber was set under mild vacuum. The sample was placed inside a borosilicate glass capillary (Hilgenberg), with a diameter of 1 mm. Data reduction was performed with the SAXSGUI program.

### Small-Angle Neutron Scattering

SANS data were obtained using the NG-7 SANS instrument at the National Institute of Standards and Technology (NIST), NIST Center for Neutron Research (NCNR).^52^ The sample-to-detector distances, *L*_sd_, were set to 1, 4, and 13 m, at a neutron wavelength, λ = 6 Å. Another configuration with lenses at *L*_sd_ = 15.3 m and λ = 8 Å was used to access low *Q*’s^53^ covering a total *Q*-range from 0.001 to 0.6 Å^–1^, where *Q* = 4π sin (θ/2)/λ, with the scattering angle, θ. A wavelength resolution of Δλ/λ = 14% was used. All data reduction into intensity, *I*(*Q*), versus momentum transfer, *Q* = |*Q⃗*|, was carried out following the standard procedures that are implemented in the NCNR macros to the Igor software package.^54^ The intensity values were scaled into absolute units (cm^–1^) using a direct beam. D_2_O as the solvent and the empty cell were measured separately. D_2_O was subtracted as the buffer background and empty cell scattering before analysis. The sample volume for each experiment was 0.45 mL. Error bars are expressed as one standard deviation.

### Neutron Spin-Echo Spectroscopy

NSE measurements were obtained using the BL15 instrument at the Spallation Neutron Source of the Oak Ridge National Laboratory, Oak Ridge, TN.^55^ Hellma quartz cells with 2 mm sample thickness were used to mount the samples. The sample volume for each experiment was 3.5 mL. Measurements were conducted using a neutron wavelength of 8 Å. The BL15 ECHODET software package was used for data reduction. Two background samples (D_2_O and APAP-saturated D_2_O) were measured separately and used for background subtraction accordingly.

## Results and Discussion

**Figure 2:**
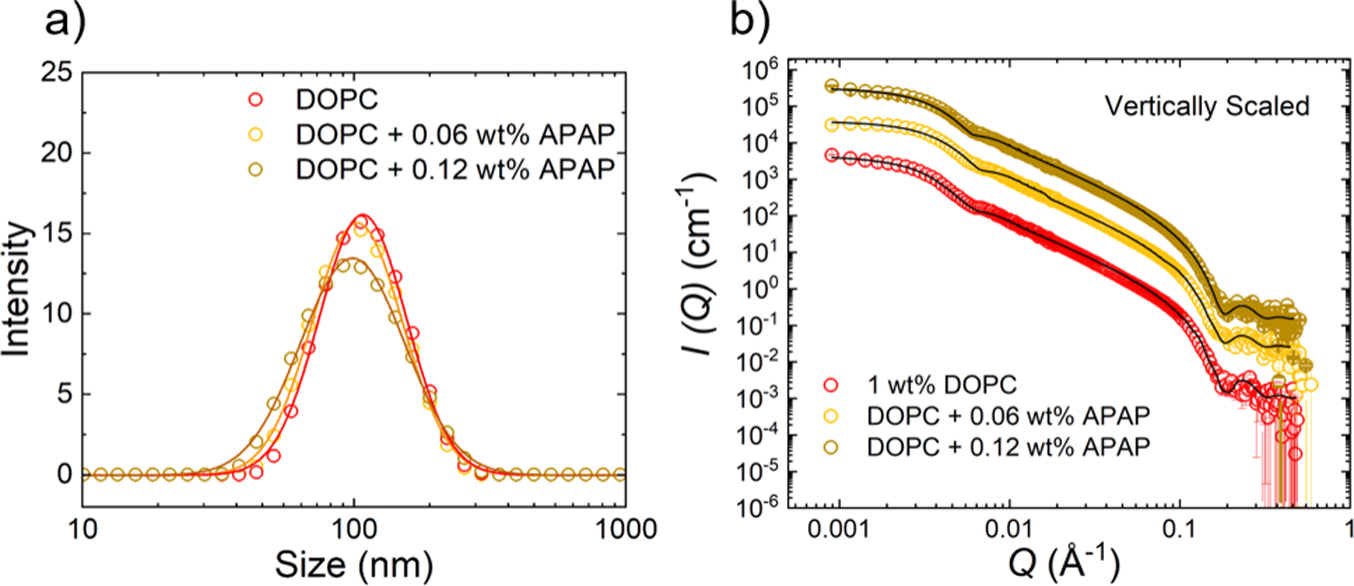
(a) DLS intensity size distribution of 1 wt % DOPC vesicles (red-empty) and DOPC with APAP with 0.06 wt % (yellow-empty) and 0.12 wt % (yellow green-empty). The solid lines show log-normal fits. (b) SANS curves for the same DOPC vesicles and DOPC/APAP vesicles. Black lines show vesicle model fits. Data are vertically scaled for better visualization. The size and other parameters obtained are listed in Table 1.

First, the structure was investigated using a combination of DLS, cryo-TEM, SANS, and SAXS. DLS results showed a slight decrease in the hydrodynamic radius of the vesicles (61.6 ± 0.2 nm, PD 37%) with increasing APAP concentrations in the lipid bilayer (APAP 0.06 wt %: 59.5 ± 0.2 nm, PD 38%, and APAP 0.12 wt %: 60.1 ± 0.3 nm, PD 44%), Table 1. Size distribution increased with the addition of APAP (Figure 2a), as reflected by the polydispersity of the vesicles calculated by log-normal distribution fits, Table 1. These results indicate an increase in size heterogeneity in the system with an increase in APAP concentration in the vesicles.

SANS data were modeled (Figure 2b) using a spherical core–shell model as described in the Supporting Information (S2). SANS data agreed with the DLS in terms of size trend (0 wt %: 51.2 ± 0.4 nm, 0.06 wt %: 43.5 ± 0.6 nm, 0.12 wt %: 46.4 ± 0.5 nm) but showed no significant change of lipid head-to-head distance of the bilayer (δ_HH_), which remained at approximately 4 nm for all three samples. However, as the aggregation number, *N*_agg_ = *V*_s_/*V*_l_, illustrates, there is a progressive decrease in the number of lipids per liposome with increasing APAP concentration (*N*_agg_ for 0 wt %: (9.3 ± 0.2) × 10^4^, 0.06 wt %: (6.7 ± 0.7) × 10^4^, and for 0.12 wt % (7.5 ± 0.5) × 10^4^, respectively. Here, *V*_s_ is the shell volume of the liposome and *V*_l_ the molar volume of the phospholipid. The observed reduction in aggregation number is almost 28% (0.06 wt % APAP) and 19% (0.12 wt % APAP) compared to the pure DOPC (0 wt % APAP). At the same time, the apparent size decrease discussed earlier leads to the surprising effect that the radius ascribed to a single lipid stays almost constant within the experimental accuracy. Eventually, a slight change of 1.06 can be calculated. Simulations on the systems APAP, DPPC, and DMPC have also revealed a virtually constant value.^27^ Hence, both simulations and experiments point to a little to no effect on the area per lipid.

We observed different polydispersities from SANS and DLS. This might be simply due to the shape anisotropies in addition to the size distributions, as indicated by Cryo-TEM (Figure 4). Similar to the results from DLS, Cryo-TEM demonstrates that a heterogeneous morphology of liposomes increases with higher concentrations of APAP. The effects of APAP on lipid vesicle deformations are especially apparent in the saturated APAP solution (Figure S4).

However, it should be noted that the polydispersity obtained from cryo-TEM images is much higher compared to SANS and DLS data. A potential explanation may be the smaller number of ∼500 particles counted in cryo-TEM compared to the statistical nature of size distributions obtained from SANS and DLS. Moreover, in SANS, the data are modeled using the statistically dominant spherical core–shell particle with uniform polydispersity for different APAP concentrations. Comparing results from cryo-TEM, DLS, and SANS suggests that although the spherical nature of the particles is statistically dominant, we might have additional particles of different shapes causing an increase in the DLS polydispersity.

**Figure 3:**
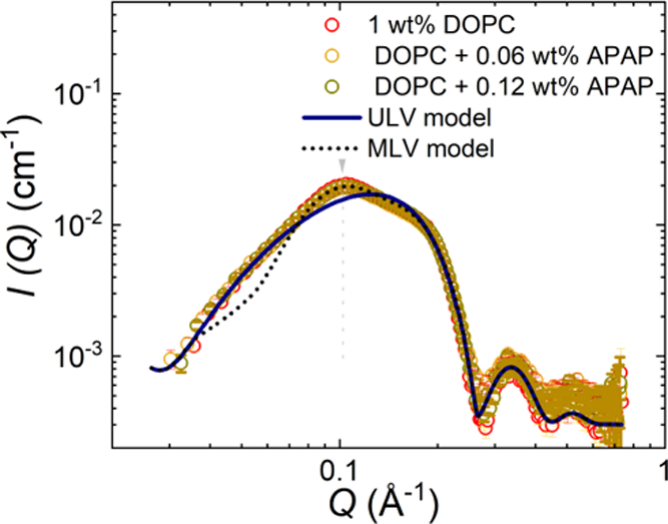
SAXS scattering data DOPC vesicles and DOPC/APAP vesicles in logarithmic scales. The data are modeled for unilamellar vesicle (ULV) (*N* = 1) (blue line) and multilamellar vesicle (MLV) models (*N* = 2) (black dashed line), respectively.

While SANS provides information on the liposome diameter, due to its resolution, SAXS can be used to obtain an accurate estimate of the lipid head and tail thickness as well as the number of bilayers. This sensitivity is achieved due to the reduced influence from vesicle form-factor over the relevant *Q*-range, higher X-ray contrast for phosphorus atoms in the lipid heads, and excellent instrumental resolution. Figure 3 illustrates SAXS diffraction patterns for 1 wt % DOPC with 0–0.12 wt % APAP concentrations.

**Figure 4:**
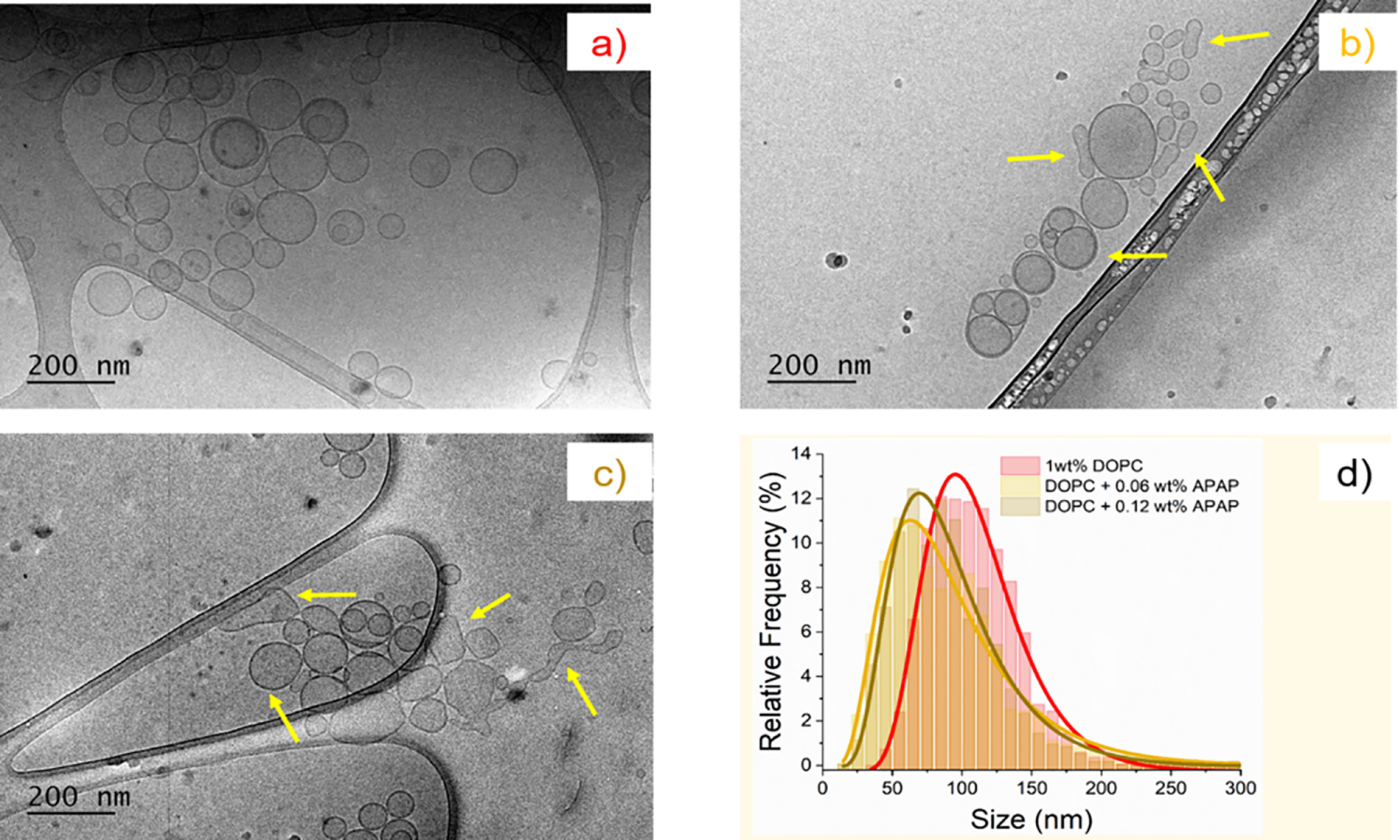
cryo-TEM images and analysis of DOPC vesicles with APAP. (a) 1 wt % pure DOPC in D_2_O, (b) 1 wt % DOPC with 0.06 wt % APAP, (c) 1 wt % DOPC with 0.12 wt % APAP, and (d) size distributions (particle analysis) for a, b, and c samples (red, yellow, and yellow-green respectively). The yellow arrows indicate vesicles departing from the original spherical state. More images and analysis details are included in the Supporting Information.

We observed only a negligible difference in the scattering pattern for different APAP concentrations. The peak at *Q* = 0.1 Å^–1^ (vertical arrow) indicates a small deviation from the simple ULV model (blue solid line), with *N* = 1 in eq 3. This peak corresponds to the evolution of the inter-bilayer structure factor peak and is modeled using an MLV model (black dashed line) described by eq 3 and in the Supporting Information, for *N* = 2 layers, where η_cp_ is kept constant at 0.01. The first-order Bragg peak given by *Q*_1_ = 0.1 Å^–1^ corresponds to a lamellar repeat distance *d* = 6.3 nm and the average value of the bilayer thickness δ_HH_ = 4.14 ± 0.5 nm; within experimental accuracy it is the same for different APAP concentrations. A Gaussian polydispersity of 22% was introduced to model *d*. The small discrepancy for *Q* < 0.07 Å^–1^ is attributed to the presence of a mixture of ULV, MLV, and overlapping vesicle structures, resulting in an apparent higher polydispersity than expected for a simple MLV model.^56^ Such a scenario can occur due to a small percentage of MLVs present in the vesicle suspension and which is evident by some of the cryo-TEM images, Figure 4 and Supporting Information.

Previous studies have shown substantial differences in SAXS profiles when small drug molecules alter the melting temperature *T*_m_ of the lipid bilayers.^57^ We assume that the relatively small changes in the SAXS profile may relate to the fluid phase (experiments are conducted well above the *T*_m_ of DOPC).

In order to visualize the morphology of the vesicles at different APAP concentrations, cryo-TEM was used. Cryo-TEM enables the visualization of phospholipid vesicles in their native state.^58^ As shown in Figure 4a, pure DOPC vesicles have a spherical morphology. The presence of occasional bilamellar vesicles confirms the slight deviation of the bilayer structure from the ULV model in SAXS analysis, which was previously discussed. From the particles counted in cryo-TEM images, over 76% were unilamellar vesicles. When it comes to the presence of APAP, deviations from the original spherical shape of the vesicles can be observed in Figure 4b,c.

Vesicles appeared more fluid-like and had irregular sphere shapes as well as occasional tubular shapes indicated by the yellow arrows. Such deviations appear to be more pronounced when the APAP concentration was increased from 0.06 to 0.12 wt %. Furthermore, overall vesicle sizes decreased in the presence of APAP. Figure 4d shows the size distribution of vesicles in each sample measured using multiple cryo-TEM images. More than 1500 size measurements were used for the size analysis using the log-normal distribution model to describe the data. The trend of size variation was similar to the DLS and SANS results previously presented in Table 1. Briefly, the sizes (radii) and polydispersities calculated for cryo-TEM samples were, 52.8 ± 0.5 nm, PD 32% (0 wt % APAP), 41.1 ± 0.6 nm, PD 52% (0.06 wt % APAP) and 42.0 ± 0.6 nm, PD 44% (0.12 wt % APAP) respectively. The polydispersity of the particles increased agreeing with results obtained by DLS and SANS. More images and details of the analysis are provided in the Supporting Information.

**Figure 5:**
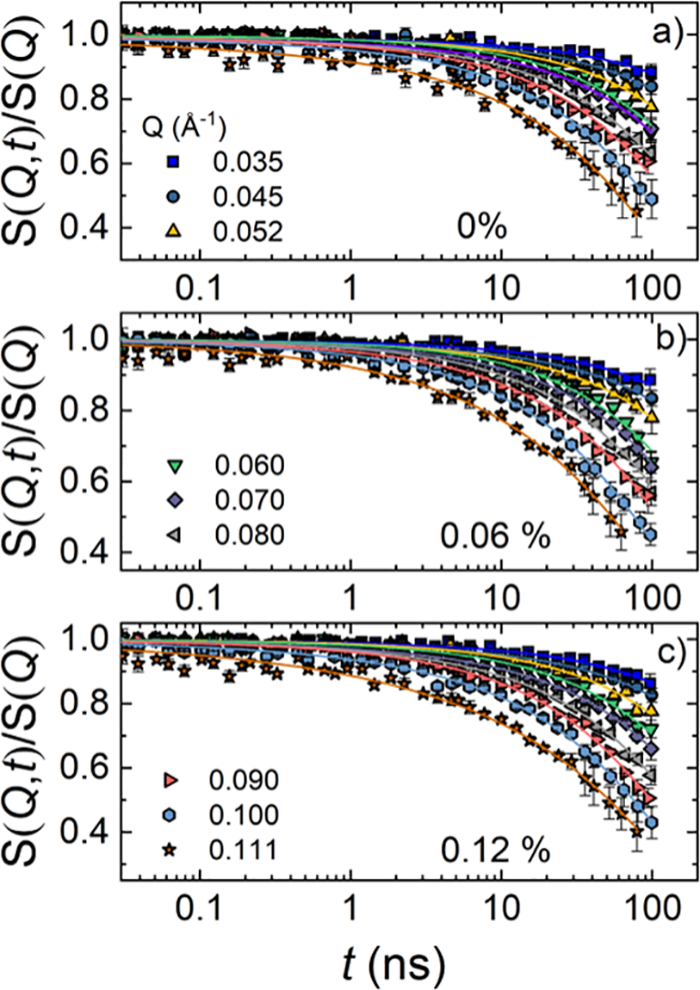
Dynamic structure factor, *S*(*Q*,*t*)/*S*(*Q*), as a function of Fourier time, *t*, for different *Q*’s, for (a) pure 1 wt % DOPC (0 wt % APAP), (b) with 0.06 wt % APAP, and (c) DOPC with 0.12 wt % APAP at room temperature. The data are analyzed using the multiplicative model.^38^

Membrane dynamics were measured by NSE spectroscopy. Data were modeled by the multiplicative model (eq 4) which includes vesicle translational diffusion, membrane fluctuations, and confined motion of lipid tails for dynamic structure factor *S*(*Q*,*t*) analysis.^38^ All experiments were conducted at room temperature. Therefore, DOPC lipids were in the fluid phase. The model agreed well with the data in the *Q* and Fourier time range used for the experiment (Figure 5). It should be noted that within the NSE time window, the translational diffusion, *D*_t_, is almost by a factor 5 slower than the ZG decay as observed from the dynamic structure factor illustrated in Figure S7 in the Supporting Information. The details of the NSE data analysis are presented in the Supporting Information.

Bending rigidity values, κ_η_, calculated from the analysis are displayed in Table 2. Briefly, for 1 wt % DOPC and 1 wt % DOPC with 0.06 and 0.12 wt % APAP, we obtained membrane rigidity values of 17.0 ± 2.0, 10.3 ± 1.0 and 9.0 ± 1.0 *k*_B_*T*, respectively. It should be noted that the small effects of MLV and fused vesicle structures as observed from SAXS and cryo-TEM images have been neglected in the calculation of the membrane rigidity from eq 6.

**Table 2:** Membrane Bending Rigidity of 1 wt % DOPC Vesicles and DOPC/APAP Vesicles from NSE Obtained from the Analysis Presented in Figure 7

1 wt % DOPC vesicles with APAP concentration wt %	bending modulus κ_η_ (*k*_B_*T*)	normalized bending rigidity 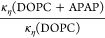
0	17.0 ± 2.0	1.0
0.06	10.3 ± 1.0	0.6
0.12	9.0 ± 1.0	0.5

**Figure 6:**
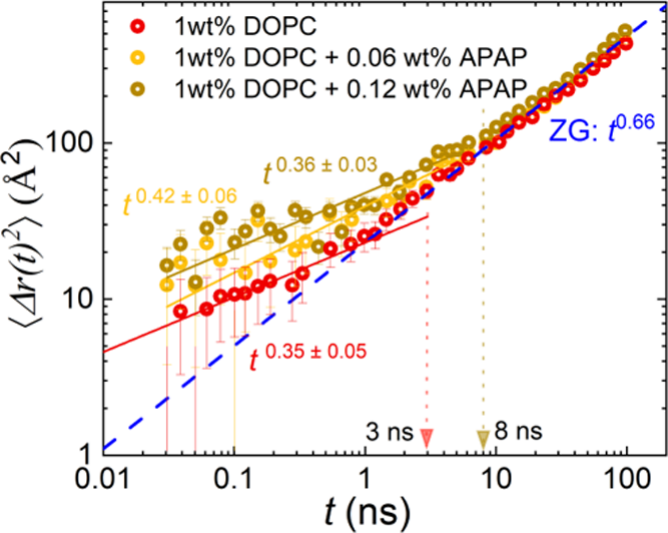
Mean square displacement, Δ*r*(*t*)^2^, vs Fourier time, t, for 1 wt % DOPC (red) and DOPC with acetaminophen (APAP): APAP 0.06 wt % (yellow) and DOPC with APAP 0.12 wt % (yellow-green). The solid lines represent the experimental power-law dependence for each sample. The blue dashed line shows the ZG *t*^0.66^ for reference. Vertical dashed lines at 1 ns and 8 ns show deviation from the ZG behavior below ∼1 ns for pure DOPC vesicles and ∼8 ns for vesicles with APAP, respectively.

Figure 6 is expressing the dynamics using the MSD, which provides information without relying on a specific model. The corresponding changes in the slope in the double-logarithmic plot or the respective power laws indicate different dynamical processes.

Within the time window of our NSE experiment, the MSD highlights two different time domains as indicated by different power laws. (1) Above 8 ns in Fourier time, results point to *t*^0.66^ dependence for all samples. Such an exponent points to a ZG region corresponding to membrane fluctuations. With the addition of APAP, the value of ⟨Δ*r*(*t*)^2^⟩ increases with the concentration, which would be consistent with a stronger fluctuation that leads to a lower bending modulus, compatible with the parameters obtained by the fit of the multiplicative model. Data representation in the double logarithmic plot hides that the MSD in this region changed by a factor of 1.03 for 0.06 wt % APAP and 1.13 for 0.12 wt % APAP compared to the pure DOPC (Supporting Information Figure S9a). (2) For low Fourier times, DOPC vesicles show a different power law, with a slope and onset that seem to change with the APAP concentration. However, within the statistical accuracy of the experimental NSE data, the results would also be compatible with an unchanged slope. This explanation is supported by our previous results on DOPC, which showed a power-law exponent of 0.26 ± 0.03.^33,39^ Considering the standard deviation, the results agree but point to caution when it comes to the deduction of a systematic change with the APAP concentration. Instead, the systematic change of the MSD with increasing APAP concentration in the short time regions seems to be statistically more significant than the change in the ZG range. In this region, MSD changes by a factor of 1.45 (0.06 wt % APAP) or 1.75 (0.12 wt % APAP) compared to the pure DOPC (Supporting Information Figure S9b). This change points to more spatial freedom for the tail motion once APAP has been added to the system. The transition to the ZG region seems to depend on the concentration of the APAP as well.

**Figure 7:**
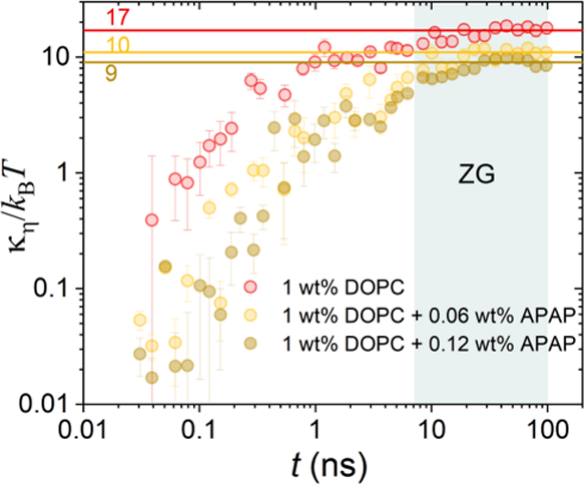
Membrane rigidity, κ_η_, divided by thermal energy, *k*_B_*T*, with the Boltzmann constant, *k*_B_, and the temperature, *T*, as a function of Fourier time *t*. The data were calculated over the NSE time window from the MSD, for 1 wt % DOPC and DOPC with APAP (0.06 wt % and 0.12 wt %) at room temperature. The calculated average values from the flat ZG region (blue) are illustrated by the horizontal lines. These lines represent the bending modulus, κ/*k*_B_*T*. Values are listed for visualization.

Considering the fast dynamics at *t* < 8 ns, we have calculated κ_η_/*k*_B_*T* using eq 6 as a function of the Fourier time over the entire NSE time window. The results for 0, 0.06, and 0.12% APAP concentrations are presented in Figure 7. The results are calculated using η = η_solvent_ and for *D*_t_ = 0.^33,38,39^ The average bending rigidity values found from the analysis within the ZG region are displayed in Figure 7 and are similar to those observed from the multiplicative model (eq 4) as displayed in Table 2.

The NSE experiments show that the incorporation of APAP in the lipid bilayer has decreased the membrane rigidity of DOPC vesicles significantly. We compared our results to existing experiments that have investigated the membrane rigidity changes with other drugs such as Ibuprofen, Aspirin, Indomethacin, and so on and demonstrated the decrease in membrane rigidities are comparable. To obtain model-independent insights, we have compared the normalized membrane rigidities. In order to see a trend with the molar fraction of drugs in the lipid membrane and decrease in membrane rigidity, the κ_η_ ratio is obtained by normalizing compared to the pure phospholipid vesicle systems used in the respective studies (Table 3).

**Table 3:** Comparison of Normalized Bending Rigidity Values of Phospholipid Vesicles in the Fluid Phase with Various Small Drug Molecules as a Function of the Molar Percentage of the Drugs in Respective Lipid Membranes

drug	lipid and conditions	molar % drug	method	normalized bending rigidity 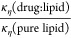	reference
no drugs	DOPC, LUV	0	NSE-multiplicative model	1	this work
Acetaminophen	DOPC, LUV	25	NSE-multiplicative model	0.6	this work
Acetaminophen	DOPC, LUV	37.5	NSE-multiplicative model	0.53	this work
Ibuprofen	DMPC	25	NSE-ZG model	0.67	(59)
Indomethacin	DMPC	25	NSE-ZG model	0.61	(59)
Aspirin	DMPC	25	NSE-ZG model	0.81	(59)
Aspirin	DMPC	36	NSE-ZG model	0.67	(60)
Salicylate	SOPC, GUVs	N/A (1 mM)	micropipette aspiration and dynamic tension spectroscopy	0.67	(19)
Ibuprofen	DMPC (pH < 2), SUV	30	NSE-ZG model	0.67	(20)
Ibuprofen	DMPC (pH > 2), SUV	30		0.47	(20)

Although the results represent different phospholipids, experimental conditions, and techniques, all phospholipids are at conditions where they are in the fluid phase, making the comparison valid. We compared our bending rigidity values with those values from other existing work and established a general trend in membrane rigidity changes induced by structurally similar drugs on phospholipid vesicles in the fluid phase. The decreasing membrane rigidity can stem from various origins. For Ibuprofen, it has been shown to be a multi-step, concentration-dependent process where the molecules get inserted into the interface region, insert and align in the lipid tail region, and disrupt membrane and induce hole formation consecutively. This is similar to the mechanism of antimicrobial peptides although structurally and chemically, they are vastly different.^13,61^ For indomethacin, researchers have shown that the drug’s association with lipids in the fluid phase is exothermic and enthalpy-driven. Specifically for DOPC, the energy release was high due to the higher spacing between acyl chains allowing more drug molecules to intercalate deeper in the hydrophobic region.^62^ Aspirin, on the other hand, has been shown to interact with the head group region and yet contribute to increased fluidity.^63^ Although all the drugs mentioned impact fluidity of the lipid membrane, there seems to be no universality of the location of these drugs in the lipid membrane. However, the severity of impact is different for drugs explored showing that each drug can have unique impacts on the lipid membrane dynamics. Furthermore, APAP interestingly did not show significant modifications in the bilayer thickness as reported for other drugs discussed. Therefore, we believe the decrease in membrane rigidity by APAP is stemming from changes in the lipid dynamics.

## Conclusions

In summary, we have investigated the concentration-dependent impact of Acetaminophen (APAP) or commonly known as paracetamol on DOPC-based LUVs in the fluid phase. We observed a slight decrease in vesicle size by DLS and SANS. Our cryo-TEM experiments illustrated further morphological changes. Vesicle shapes became more and more irregular with the increasing concentration of APAP, which might be an explanation of the increasing polydispersity of the vesicle populations observed by DLS. We observed a decrease in aggregation number compared to the pure DOPC vesicles. Surprisingly, we did not observe much change in the bilayer thickness. However, we observed a stronger change of the mean square displacement of the lipid tail motion. This was only accessible since we expanded measurements in the short time scale (below 8 ns). It seems that the space explored by the lipid tails increases by a factor of 1.45 (for 0.06 wt % APAP) and 1.75 (for 0.12 wt % APAP) compared to DOPC without the drug. This is supported by the fact that with the incorporation of APAP, we observed a substantial reduction in membrane rigidity, by almost 50%. However, the simulations by Nademi et al. also observed a virtually constant area per lipid but a change of the lateral diffusion.^27^ In the case of Nademi, the authors calculated the mean square displacement of the whole lipid while our experiments see mainly the lipid tail motions since neutrons measure the protons and most of the protons are in the tail. The simulations find a substantial decrease, which the authors ascribe to drug molecules entering the space between the lipids. Our results also point to drug molecules increasing the space between lipids, which would explain the larger MSD of the tails. As the distance between the molecules determines the molecular interactions, the reduced bending modulus seems to be a direct consequence of the increased spatial freedom. Again, we point to the surprising area per lipid from the SANS experiments, which is virtually unchanged (at most changed by a factor of 1.06). Therefore, despite little changes in the static structure, strong changes of the tail motion and bending elasticity seem to be a consequence of drug molecules penetrating the free space between the lipids.

Since APAP toxicity has been related to multiple cellular functions such as oxidative stress, lipid peroxidation,^62^ and so on, the changes in membrane dynamics and fluidity may be directly or indirectly connected to these cellular functions and hence should not be ignored. This also opens up therapeutic avenues to explore the usage of clinically well-established drugs such as APAP in cancer therapeutic drug delivery systems as an agent to manipulate membrane rigidity.^63^

## Supporting Information Available

Physicochemical details of APAP, Cryo-TEM additional images and analysis details, SAXS data from CAMD—lab X-ray source, NSE data modeling using the ZG model, impact of translational diffusion on NSE data, and ZG decay rate Γ_Q_ variation with and without the translational diffusion of the vesicles (PDF)

## Notes

Certain trade names and company products are identified in order to specify adequately the experimental procedure. In no case does such identification imply recommendation or endorsement by the National Institute of Standards and Technology, nor does it imply that the products are necessarily the best for the purpose. This report was prepared as an account of work sponsored by an agency of the United States Government. Neither the United States Government nor any agency thereof, nor any of their employees, makes any warranty, express or implied, or assumes any legal liability or responsibility for the accuracy, completeness, or usefulness of any information, apparatus, product, or process disclosed or represents that its use would not infringe privately owned rights. Reference herein to any specific commercial product, process, or service by trade name, trademark, manufacturer, or otherwise does not necessarily constitute or imply its endorsement, recommendation, or favoring by the United States Government or any agency thereof. The views and opinions of the authors expressed herein do not necessarily state or reflect those of the United States Government or any agency thereof.
