# Supplementary material for: Acetaminophen Interactions with Phospholipid Vesicles Induced Changes in Morphology and Lipid Dynamics
Source: Langmuir. 2021 Jul 30;37(31):9560–70. doi: 10.1021/acs.langmuir.1c01458 (PMC8359007; doi:10.1021/acs.langmuir.1c01458)
Supplement: Supplementary file 1 — la1c01458_si_001.pdf [file la1c01458_si_001.pdf]

Supporting Information for Acetaminophen Interactions with Phospholipid Vesicles Induced Changes in Morphology and Lipid Dynamics

Judith U. De Mel[1*], Sudipta Gupta[1*], Sydney Harmon[2], Laura Stingaciu[3], Eric W. Roth[4], Miriam Siebenbuerger[5] Markus Bleuel[6], Gerald J. Schneider[1,7*]

[1]Department of Chemistry, Louisiana State University, Baton Rouge, LA 70803, USA

[2]Department of Chemistry, Colorado School of Mines, Golden, CO 80401, USA

[3]Neutron Sciences Directorate, Oak Ridge National Laboratory (ORNL), POB 2008, 1 Bethel Valley Road, Oak Ridge, TN 37831, USA

[4]Department of Materials Science and Engineering and NUANCE Center, Northwestern University, 2220 Campus Drive, Evanston, Illinois 60208, USA

[5]Center of Advanced Microstructures and Devices, Louisiana State University, 6980 Jefferson Highway, Baton Rouge, LA 70806, USA

[6]NIST Center for Neutron Research, National Institute of Standards and Technology, Gaithersburg, MD 20899-8562, USA

[7]Department of Physics & Astronomy, Louisiana State University, Baton Rouge, LA 70803, USA

Correspondence should be addressed to*: demeljudith@gmail.com, g.sudipta26@gmail.com, gjschneider@lsu.edu

Table of Contents

Table S1 Acetaminophen Physicochemical Data — Page S2
SANS Data Modeling — Page S2
SAXS Data Modeling — Page S3
Figure S1 Additional cryo TEM images of pure DOPC Vesicles — Page S4
Figure S2 Additional cryo TEM images of DOPC vesicles with 0.06 wt% APAP — Page S5
Figure S3 Additional cryo TEM images of DOPC vesicles with 0.12 wt% APAP — Page S5
Figure S4 Additional cryo TEM images of DOPC vesicles in APAP solution — Page S6
Log-Normal Distribution — Page S6
Figure S5 SAXS data compared for formulation Batch1 and Batch 2 (CAMD) — Page S7
Figure S6 NSE data modeling using ZG model for different APAP concentrations — Page S7
Figure S7 Effect of translational diffusion on the NSE data — Page S7
Figure S8 Variation of ZG decay rate Γ_Q for different APAP concentrations — Page S8
Figure S9 Relative MSD in low Fourier times and ZG region — Page S8
References — Page S8

Table S1 Acetaminophen Physicochemical Data

Chemical/Physical Property	
Chemical Formula and Molecular Weight	$C_8H_9NO_2$, 151.1626 g mol^{-1}
pK_a	9.5
Melting Point	168-172 °C
Polymorphic forms isolatable	Form I (monoclinic), Form II (orthorhombic)
(Sources)[1, 2]	

SANS Data Modeling

The 1D scattering pattern is given by:

$$P(Q,R,t,\Delta\rho) = \frac{\phi A^2(Q)}{V(r_3) - V(R_c)} \qquad \text{(S1)}$$

with ϕ is the lipid volume fraction.

The scattering contribution from three different shells are given by

$$A^2(Q) = A_1^2 + A_2^2 + A_3^2 + A_{12} + A_{23} + A_{13} \qquad \text{(S2)}$$

with $A_{12} = A_1A_2$, $A_{12} = A_2A_3$, and $A_{13} = A_1A_3$ are the cross-terms for inner-head-tail, tail-outer-head and the two outer lipid head layers, respectively.

For a spherical Bessel's function $j_1(x) = \frac{\sin(x) - x\cos(x)}{x^2}$, and volume $V(r) = \frac{4}{3}\pi r^3$ we have individual scattering amplitudes as:

$$A_1^2 = \left(\rho_{head} - \rho_{\text{solv}}\right)^2 \left[3V(r_1)\frac{j_1(Qr_1)}{Qr_1} - 3V(R_c)\frac{j_1(QR_c)}{QR_c}\right]^2$$

$$A_2^2 = \left(\rho_{tail} - \rho_{\text{solv}}\right)^2 \left[3V(r_2)\frac{j_1(Qr_2)}{Qr_2} - 3V(r_1)\frac{j_1(Qr_1)}{Qr_1}\right]^2$$

$$A_3^2 = \left(\rho_{head} - \rho_{\text{solv}}\right)^2 \left[3V(r_3)\frac{j_1(Qr_3)}{Qr_3} - 3V(r_2)\frac{j_1(Qr_2)}{Qr_2}\right]^2$$

$$A_{12} = 2(\rho_{tail} - \rho_{\text{solv}})(\rho_{head} - \rho_{\text{solv}})\left[3V(r_1)\frac{j_1(Qr_1)}{Qr_1} - 3V(R_c)\frac{j_1(QR_c)}{QR_c}\right]\left[3V(r_2)\frac{j_1(Qr_2)}{Qr_2} - 3V(r_1)\frac{j_1(Qr_1)}{Qr_1}\right] \qquad \text{(S3)}$$

$$A_{23} = 2(\rho_{tail} - \rho_{\text{solv}})(\rho_{head} - \rho_{\text{solv}})\left[3V(r_2)\frac{j_1(Qr_2)}{Qr_2} - 3V(r_1)\frac{j_1(Qr_1)}{Qr_1}\right]\left[3V(r_3)\frac{j_1(Qr_3)}{Qr_3} - 3V(r_2)\frac{j_1(Qr_2)}{Qr_2}\right]$$

$$A_{13} = 2(\rho_{head} - \rho_{\text{solv}})(\rho_{head} - \rho_{\text{solv}})\left[3V(r_1)\frac{j_1(Qr_1)}{Qr_1} - 3V(R_c)\frac{j_1(QR_c)}{QR_c}\right]\left[3V(r_3)\frac{j_1(Qr_3)}{Qr_3} - 3V(r_2)\frac{j_1(Qr_2)}{Qr_2}\right]$$

Here, $r_1 = R_c + t_{head}$, $r_2 = R_c + t_{head} + t_{tail}$, $r_3 = R_c + 2t_{head} + t_{tail}$. The membrane thickness of the bilayer from SANS is given by, $\delta_{HH}(SANS) = 2t_{head} + t_{tail}$. For DOPC we used the neutron scattering length density (NSLD) of the hydrocarbon tail, $\rho_{\text{tail}} = -2.08 \times 10^9$ cm^{-2}, and for phosphatidylcholine (PC) head group, $\rho_{\text{head}} = 1.73 \times 10^{10}$ cm^{-2} has been used.[3] For D_2O the NSLD of the solvent of, $\rho_{\text{solv}} = 6.36 \times 10^{10}$ cm^{-2} has been used. Each shell thickness and scattering length density is assumed to be constant for the respective shell.

SAXS Data Modeling

SAXS scattering intensity from random lamellar sheets consisting of lipid heads and tails of thicknesses are δ_H and δ_T, respectively is given by

$$\frac{d\Sigma}{d\Omega}(Q)_{SAXS} = 2\pi\frac{\phi P(Q)S(Q)}{Q^2 d} \quad \text{(S4)}$$

with particle volume fraction, ϕ, and the lamellar repeat distance, *d*. The form factor is given by:

$$P(Q) = \frac{4}{Q^2}\left[\Delta\rho_{\text{H}}\{\sin(Q(\delta_H+\delta_T)) - \sin(Q\delta_T)\} + \Delta\rho_{\text{T}}\sin(Q\delta_T)\right]^2 \quad \text{(S5)}$$

The scattering contrasts for the head and tail are $\Delta\rho_{\text{H}}$ and $\Delta\rho_{\text{T}}$, respectively. The corresponding thicknesses are δ_H and δ_T, respectively. The head to head bilayer thickness is given by, $\delta_{HH}(SAXS) = 2(\delta_H+\delta_T)$. For unilamellar structure, $N = 1$, the repeat distance in equation 6 is given by the membrane thickness, δ_{HH}. The Caille structure factor is given by

$$S(Q) = 1 + 2\sum_{n=1}^{N-1}\left(1 - \frac{n}{N}\right)\cos(Qdn)\exp\left(-\frac{2Q^2d^2\alpha(n)}{2}\right) \quad \text{(S6)}$$

with the number of lamellar plates, N ($N > 1$), and the correlation function for the lamellae, $\alpha(n)$, defined by

$$\alpha(n) = \frac{\eta_{\text{cp}}}{4\pi^2}(\ln(\pi n) + \gamma_E) \quad \text{(S7)}$$

with $\gamma_E = 0.57721$ the Euler's constant. The elastic constant for the membranes is expressed in terms of the Caille parameter, $\eta_{\mathrm{cp}} = \frac{{Q_1}^2 k_B T}{8\pi\sqrt{(\kappa_c \bar{B})/\delta_{HH}}}$, where κ_c and $\bar{B}$ are the bending elasticity and the compression modulus of the membranes. Here $\bar{B}$ is associated with the interactions between the membranes. The position of the first-order Bragg peak is given by Q_1, whereas k_B is the Boltzmann's constant and T the absolute temperature. A Gaussian distribution function includes thickness polydispersity for d, δ_H and δ_T.

Additional Cryo-TEM Images

(Images S1-S3 were obtained at the Northwestern University and S4 at Tulane University)

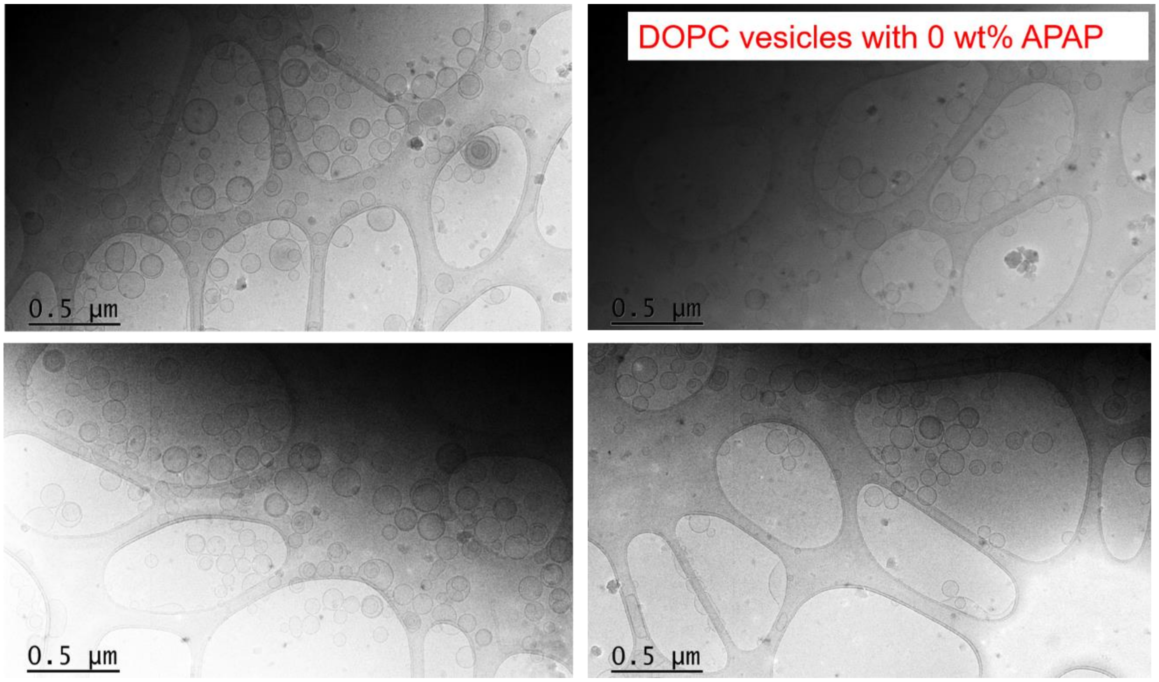

Figure S1. Additional Cryo TEM images of DOPC phospholipid vesicles without incorporated Acetaminophen (APAP). The spherical shape is maintained.

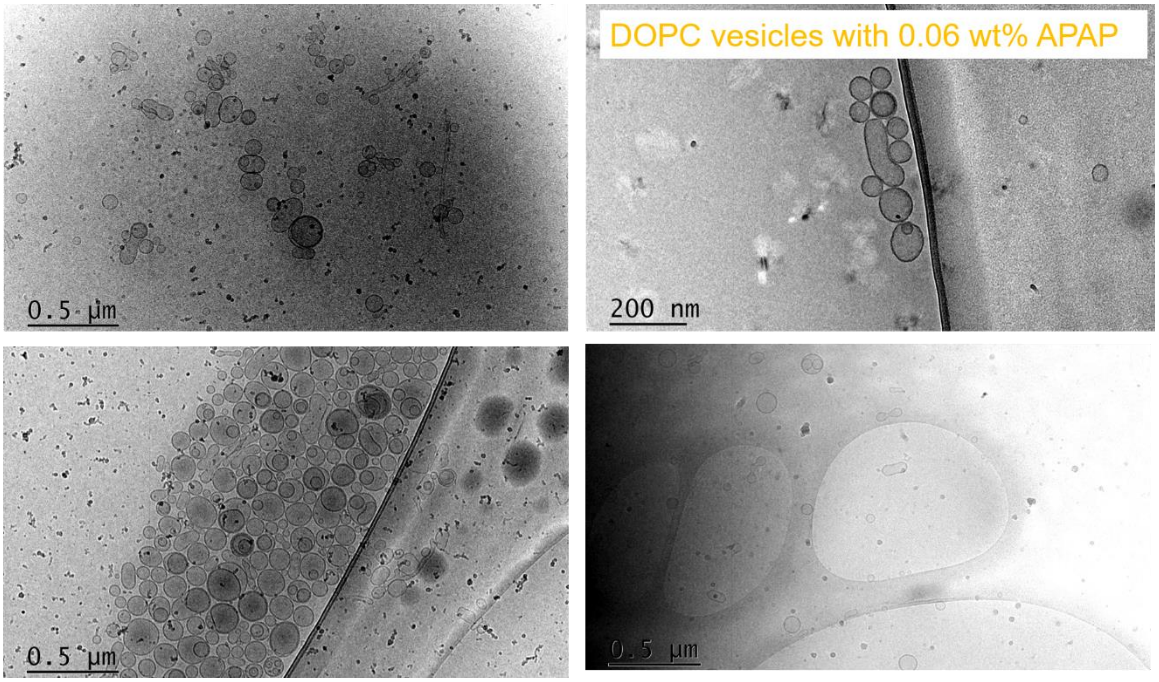

Figure S2. Additional Cryo TEM images of DOPC phospholipid vesicles with 0.06 wt% incorporated Acetaminophen (APAP). The spherical shape is deformed.

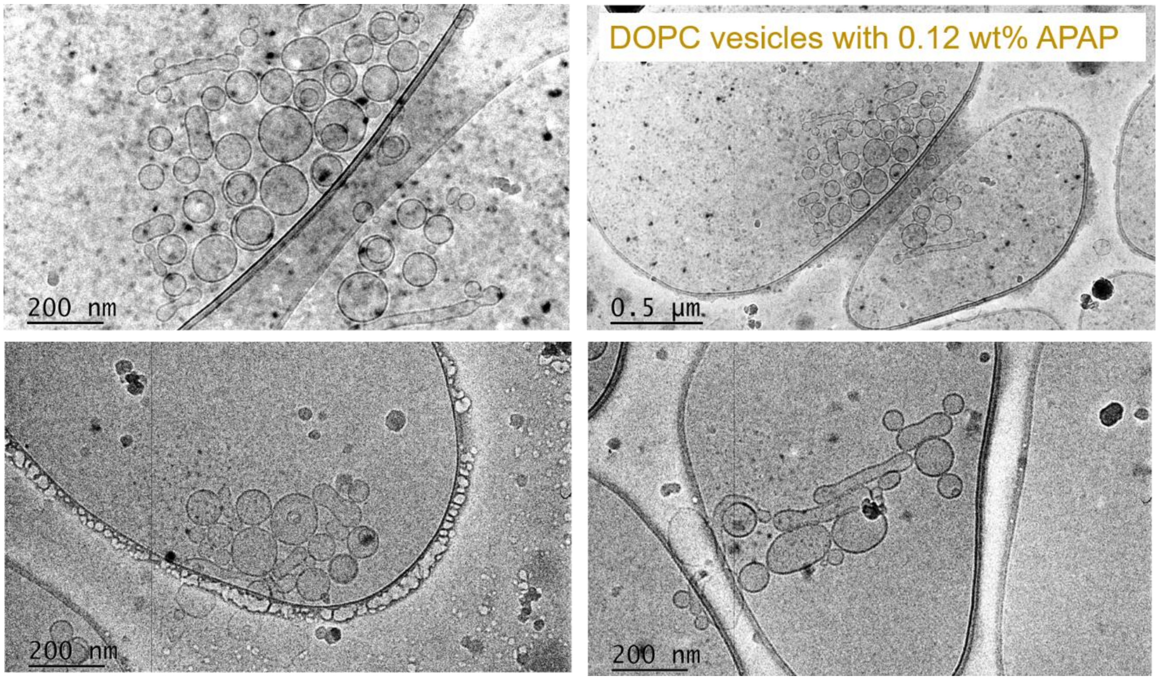

Figure S3. Additional Cryo TEM images of DOPC phospholipid vesicles with 0.12 wt% incorporated Acetaminophen (APAP). The morphology of vesicles is irregular and polydisperse.

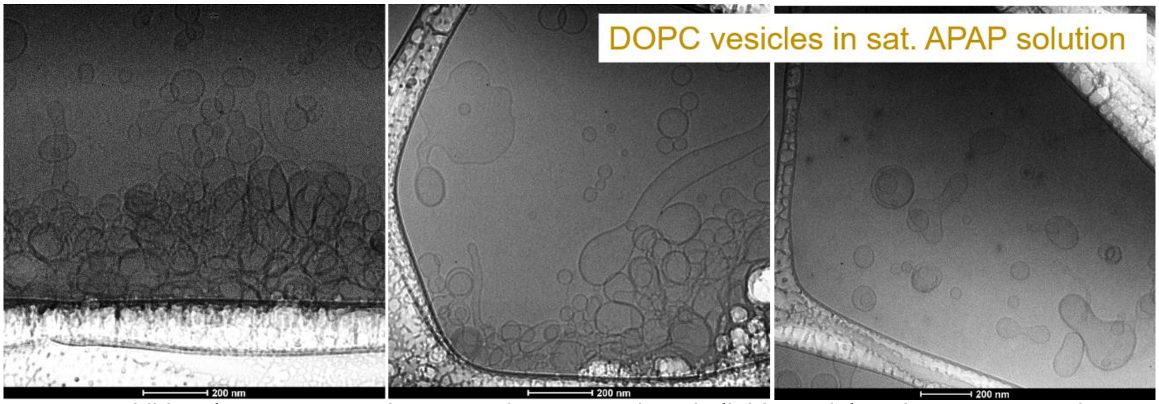

Figure S4. Additional Cryo TEM images of DOPC phospholipid vesicles in a saturated aqueous Acetaminophen (APAP) solution. The vesicle morphologies are deformed.

All results on the size and size distribution presented in the main text were analyzed using the ImageJ software. The parameterization was obtained by a log-normal distribution, given by

$$s(r) = \frac{1}{\sigma r\sqrt{2\pi}} \exp\left(-\frac{[\ln(r/R_{\text{median}})]^2}{2\sigma^2}\right) \quad \text{(S8)}$$

where R_{median} refers to the radius of the particle and, σ is the standard deviation representing the polydispersity, $\sigma \times 100$ %.

Since data were obtained for two formulation batches (Batch 1: DLS, SANS, NSE, SAXS at Stanford Synchrotron and Batch 2: Cryo TEM, SAXS at LSU CAMD synchrotron), SAXS data were compared to test reproducibility. Figure S5 shows LSU CAMD SAXS data (left) compared to the SAXS data obtained at Stanford Synchroton (right) which is reproduced in Figure 3 from MS.

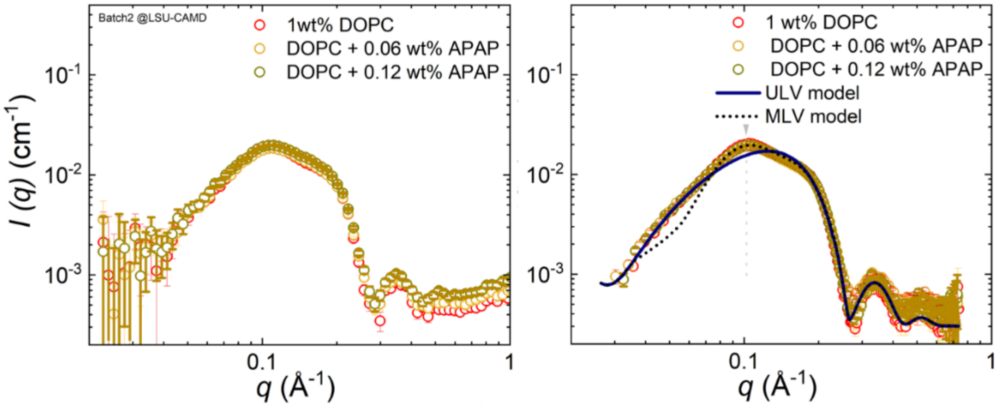

Figure S5. SAXS data comparison of DOPC vesicle formulations Batch 2 and 1. Data are reproducible

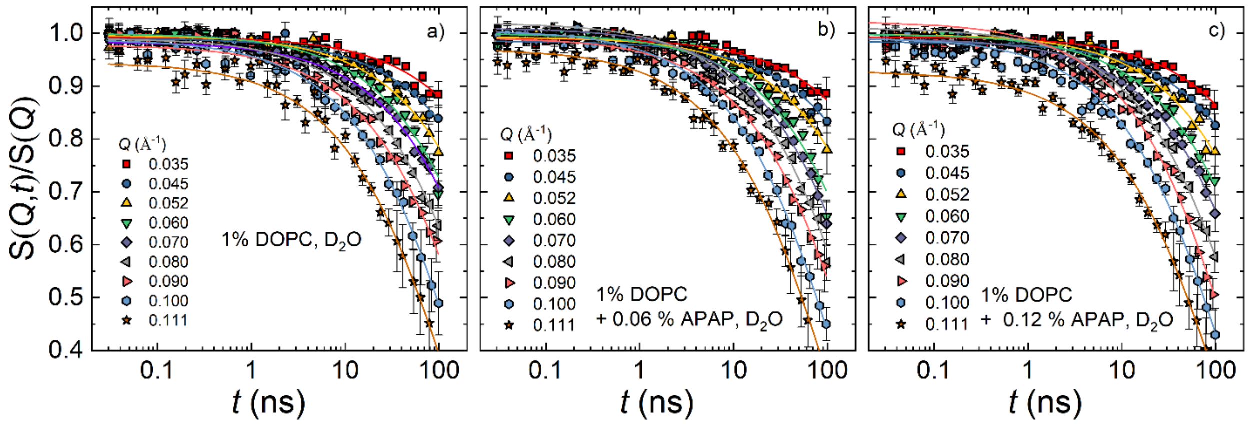

Figure S6. Dynamic structure factor, $S(Q,t)/S(Q)$, as a function of Fourier time, t, for different Q's, for a) pure 1wt% DOPC, b) with 0.06 wt% Acetaminophen (APAP) and c) DOPC with 0.12 wt% APAP at Room Temperature. The data are modeled using Zilman-Granek (ZG) model.

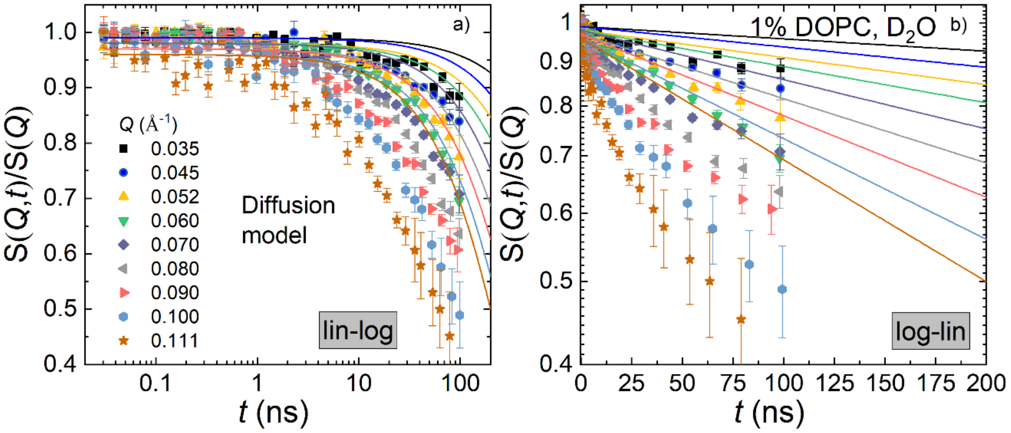

Figure S7. Illustrating the effect of translational diffusion, D_t on the dynamic structure factor, $S(Q,t)/S(Q)$, for 1 % DOPC and 0.0% Acetaminophen (APAP) concentrations in D_2O, in (a) linear-logarithmic, and (b) logarithmic-linear scale. The solid lines represent the decay corresponding to translational diffusion, $exp(-Q^2 D_t t)$. The diffusion coefficient, D_t, obtained from dynamic light scattering has been used.

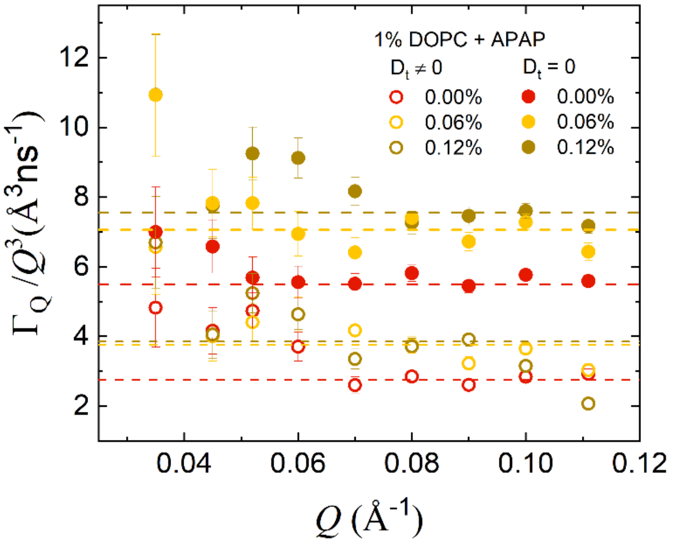

Figure S8. Variation of ZG decay rate Γ_Q , as a function of Q for different Acetaminophen (APAP) concentrations. The results were calculated by (i) considering translational diffusion, $D_t \neq 0$, and (ii) not considering translational diffusion, $D_t = 0$, affecting the ZG decay rate.

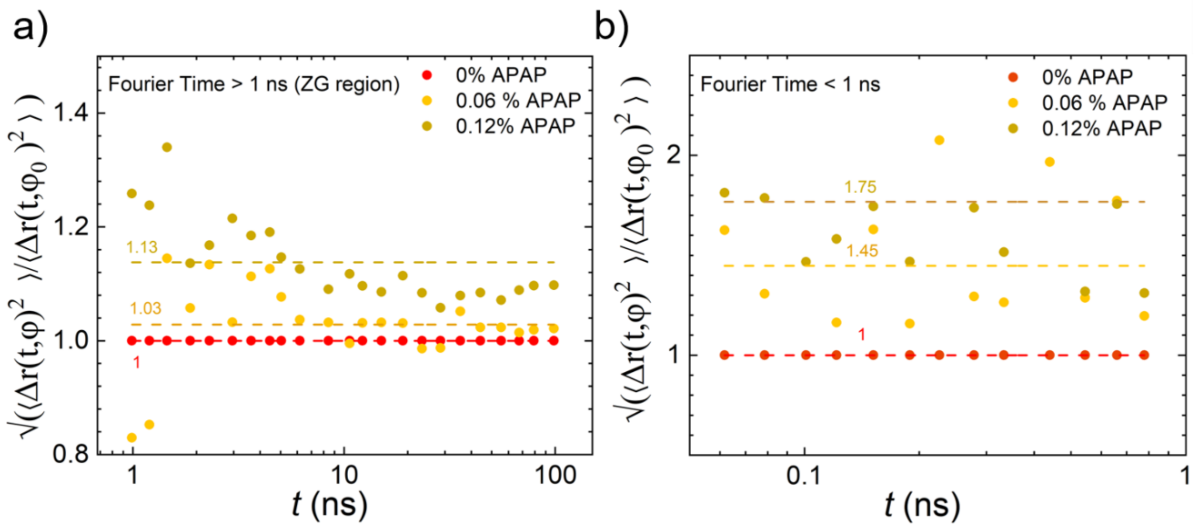

Figure S9. Variation of relative displacement $\sqrt{\frac{\langle \Delta r(t,\varphi)^2 \rangle}{\langle \Delta r(t,\varphi_0)^2 \rangle}}$ as a function of Fourier time t for different Acetaminophen (APAP) concentrations a) ZG region (1- 100 ns)and b) Low Fourier times (below 1 ns)

References

1. Xu, F.; Sun, L. X.; Tan, Z. C.; Liang, J. G.; Zhang, T., Adiabaticcalorimetry and thermal analysis on acetaminophen. *Journal of Thermal Analysis and Calorimetry* **2006,** *83* (1), 187-191.
2. Peterson, M. L.; Morissette, S. L.; McNulty, C.; Goldsweig, A.; Shaw, P.; LeQuesne, M.; Monagle, J.; Encina, N.; Marchionna, J.; Johnson, A.; Gonzalez-Zugasti, J.; Lemmo, A. V.; Ellis, S. J.; Cima, M. J.; Almarsson, Ö., Iterative High-Throughput Polymorphism Studies on Acetaminophen and an Experimentally Derived Structure for Form III. *Journal of the American Chemical Society* **2002,** *124* (37), 10958-10959.

3. Benedetto, A.; Heinrich, F.; Gonzalez, M. A.; Fragneto, G.; Watkins, E.; Ballone, P., Structure and Stability of Phospholipid Bilayers Hydrated by a Room-Temperature Ionic Liquid/Water Solution: A Neutron Reflectometry Study. *The Journal of Physical Chemistry B* **2014,** *118* (42), 12192-12206.
